# Scheduling of Remote Monitoring for Peritoneal Dialysis Patients

**DOI:** 10.3390/jcm13020406

**Published:** 2024-01-11

**Authors:** Grazia Maria Virzì, Niccolò Morisi, Sabrina Milan Manani, Ilaria Tantillo, José David Gonzàlez Barajas, Bladimir Diaz Villavicencio, Claudia Castiglione, Gaetano Alfano, Gabriele Donati, Monica Zanella

**Affiliations:** 1 Department of Nephrology, Dialysis and Transplantation, San Bortolo Hospital, 36100 Vicenza, Italy; graziamaria.virzi@gmail.com (G.M.V.); ilaria.tantillo@aulss8.veneto.it (I.T.); monica.zanella@aulss8.veneto.it (M.Z.); 2 IRRIV—International Renal Research Institute Vicenza Foundation, 36100 Vicenza, Italy; niccomorisi@gmail.com (N.M.); jdavidglez93@gmail.com (J.D.G.B.); blad.villavicencio@hotmail.com (B.D.V.); 3 Surgical, Medical and Dental Department of Morphological Sciences, Section of Nephrology, University of Modena and Reggio Emilia, 41121 Modena, Italy; gaetano.alfano@unimore.it (G.A.); gabriele.donati@unimore.it (G.D.); 4 Departiment of Nephrology, University of Guadalajara Health Sciences Center, Guadalajara 44100, Mexico; 5 Department of Medicine, Section of Nephrology, University of Verona, 37129 Verona, Italy; claudia.castiglione@univr.it

**Keywords:** peritoneal dialysis, remote patient monitoring (RPM), workflow, automated peritoneal dialysis (APD)

## Abstract

Peritoneal dialysis (PD) is performed as a home-based treatment and in this context, telemedicine has been proven helpful for improving clinicians’ surveillance and maintaining PD patients in their home setting. The new e-health devices make remote patient monitoring (RPM) for automated peritoneal dialysis (APD) treatment possible, evaluating the data at the end of every treatment and adapting the prescription at distance if necessary. This paper aims to share a method for improving clinical surveillance and enabling PD patients to receive their treatment at home. In the present case series, we delineate the clinical protocol of the Vicenza PD Center regarding patient characteristics, timing, and the purpose of the APD-RPM. We present the Vicenza PD Center’s experience, illustrating its application through three case reports as exemplars. Telemedicine helps to carefully allocate healthcare resources while removing the barriers to accessing care. However, there is a risk of data overload, as some data might not be analyzed because of an increased workload for healthcare professionals. A proactive physician’s attitude towards the e-health system has to be supported by clinical instructions and legislative rules. International and national guidelines may suggest which patients should be candidates for RPM, which parameters should be monitored, and with what timing. According to our experience, we suggest that the care team should define a workflow that helps in formulating a correct approach to RPM, adequately utilizing resources. The workflow has to consider the different needs of patients, in order to assure frequent remote control for incident or unstable patients, while prevalent and stable patients can perform their home treatment more independently, helped by periodic and deferred clinical supervision.

## 1. Introduction

Telehealth is becoming a regular part of clinical practice, complementing the traditional in-person healthcare system in various specialties, including cardiology, neurology, radiology, and more. Specifically, remote patient monitoring (RPM) has shown promise in the management of chronic diseases such as diabetes, chronic obstructive pulmonary disease (COPD), and heart failure [1]. In the work of Su et al. [2], there is a clear representation of the remote patient monitoring (RPM) protocol for diabetic patients in Nebraska. The monitoring system checks biometric data daily, provided by patients. The data include blood pressure, weight, and glucose levels. Subsequently, a trained nurse makes phone calls to patients at least once a week to monitor alerts indicated by the system and to provide services such as assessing medication adherence, offering nutritional counseling, measuring weight, and supporting disease self-management. The ALTITUDE study [3] investigates outcomes for patients with implantable cardioverter-defibrillator (ICD) and cardiac resynchronization therapy–defibrillator (CRT-D) devices outside of clinical trials, focusing on mortality following device implantation and shock therapy. The analysis, involving a large cohort from a single manufacturer, compares outcomes between patients monitored in device clinic settings and those regularly transmitting remote data. The results show that the 1- and 5-year survival rates are higher for patients receiving RPM follow-up compared to those monitored only in device clinics. In the work of Cruz et al. [4], RPM was valuable for COPD patients. In their review, two studies showed a decrease in exacerbations (*p* < 0.05) and a significant improvement in health-related quality of life (SMD = −0.53; 95% CI = −0.97–−0.09; *p* = 0.019). The findings suggest that home telemonitoring reduces respiratory exacerbations and hospitalizations while enhancing quality of life.

Furthermore, the potential of RPM was extensively explored during the COVID-19 pandemic to alleviate the strain on the healthcare system. Aalam et al. [5], for instance, elucidated a straightforward RPM workflow for COVID-19 outpatients. In this protocol, all outpatients are required to complete a daily questionnaire regarding their health status. Physicians then contact patients who have not responded to the questionnaire and based on the patient’s responses and a system-generated flag, a decision is made whether they require a telemedicine appointment or admission to the hospital. In conclusion, RPM has proven to be effective in overseeing both chronic illnesses and acute conditions. The utilization of RPM technologies has demonstrated significant benefits in the continuous management and timely intervention of patients with long-term health concerns, as well as in efficiently monitoring and responding to acute health situations. This dual capability underscores the versatility and success of RPM as a valuable tool in modern healthcare, offering a comprehensive approach to enhance patient care across a spectrum of medical needs.

Telehealth is also used within various areas of nephrology, in particular in the field of dialysis.

Peritoneal dialysis (PD) is performed as a home-based treatment; in this context, telemedicine has been proven to improve clinicians’ surveillance and help maintain patients in their home setting [6,7].

Thanks to digital health information technology, wearable blood pressure monitors or weight scales can be connected to a modem that sends the patient’s data to the hospital. In this way, clinicians can evaluate frequent measurements of clinical parameters, both routinely and as needed [8]. Consequently, when clinical management requires a therapy adjustment, the physicians perform a televisit, as a delivery of health care services, where distance is a critical factor. As suggested by the World Health Organization (WHO), all healthcare professionals will use information and communication technologies for the exchange of valid information for diagnosis, treatment, and prevention of disease and injuries [9].

Over the last couple of years, automated PD (APD) has been performed using a cycler machine that, through electronic devices, sends the dialysis data to the PD center. Previously, the PD program and the treatments were stored on an electronic card, and with this so-called “card system” the patients or their caregivers had to bring the card to the PD center for review.

The new e-health devices make it possible to remotely monitor the APD treatment, evaluate the data at the end of every treatment, and adapt the prescription at a distance if necessary [10,11].

In the context of this paper, we provide a comprehensive description of our strategy for implementing RPM in clinical practice. Our objective is not only to elucidate the intricacies of the RPM clinical protocol but also to propose a well-defined workflow that ensures both a robust patient follow-up and an efficient organization of the care team. Furthermore, to offer a practical illustration of the protocol’s application, we present three insightful case reports sourced from the experiences of the Vicenza Center. This case series provides illuminating exemplars, demonstrating the real-world efficacy of our proposed strategy and providing valuable insights into its successful implementation. By presenting tangible examples and case series data, we aim to facilitate a deeper understanding of how the RPM protocol can be seamlessly integrated into clinical practice, ultimately contributing to improved patient outcomes and enhanced care team coordination.

## 2. Methods

### 2.1. APD Program in Vicenza, Italy, PD Center

In the Vicenza PD Center, APD is performed using two different software systems, integrated with an internet connection. Using this system, clinicians can remotely monitor the dialysis treatments and modify the APD prescription without the need to bring the card to the PD center (Sleep-safe Harmony with Patient Card Unicard Reader, Fresenius Medical Care, Bad Homburg, Germany, and ClariaTM, Baxter Healthcare, Deerfield, IL, USA). Moreover, the patients are monitored by physicians through in-person visits (monthly regular controls and clinically motivated urgent visits). In addition, dedicated PD nurses support PD patients with at-home scheduled visits and/or urgent visits, if required.

We scheduled APD-RPM performed by physicians and nurses for the incident and prevalent patients, as reported in Table 1. In our APD-RPM clinical model, we divide patients into incident and prevalent patients. We defined incident patients as those within the first 15 days from the initiation of PD. After the first fifteen days of PD, the patients transitioned to the prevalent phase. Moreover, patients were split into stable and unstable for better follow-up, management, and clinical adequacy for each clinical condition. In the case of incident and unstable patients, the nurses monitored all the dialysis treatments in the telemedicine platform, then once a week. In the case of prevalent patients, the check was routinely carried out once a week.

Through RPM, we gathered crucial data, including catheter flow rates with drain and fill times, filling and draining volumes with ultrafiltration, total dialysis time for each cycle, alarms occurring during cycles, and the patient’s interactions with the machine. For each patient, nurses spent between five and ten minutes evaluating the data. Furthermore, they reported it to the physicians when the dialysis was not well performed—for example, if they found more than ten alarms per treatment, or if the patient performed more than three bypasses, or when the ultrafiltration was less than prescribed, or when certain treatments had not been performed. There were no clear criteria for determining when the nurse should alert the doctor regarding a monitored patient. The system aids in this regard through potential alarm notifications or reports. Due to specific nursing training in PD, some events can be managed by the nursing team without direct intervention from the doctor but by simply informing them of the situation. Moreover, the physicians and the nurses checked the treatments in the platform whenever the patients called the PD center reporting a dialytic problem. After the platform evaluation, the physicians could remotely change the dialysis prescription if needed and give the patient some suggestions by phone, avoiding an unscheduled hospital visit if the clinical conditions were stable. Meanwhile, if the clinical conditions were unstable, such as in case of overhydration, peritonitis, or catheter dysfunction, the patient was invited to come to the hospital. In addition, in cases of non-compliance, such as treatments not being performed or more than three bypasses taking place, an in-person visit was required. Similarly, when the patient returned home after the solution of an acute episode, daily remote monitoring was scheduled for incident patients.

### 2.2. Clinical Case 1

MS is a 67-year-old woman who suffers from chronic kidney disease (ESKD) caused by an aggressive form of IgA-nephropathy, which is being monitored by our center in Vicenza. In June 2022, she needed to initiate peritoneal dialysis (PD) due to the progression of her disease to ESKD, and a PD catheter was successfully placed on June 20th without any complications. Following in-hospital nurse training, Mrs. MS commenced the APD program at home using Sleep-safe Harmony and the Patient Card Unicard Reader (Fresenius Medical Care, Bad Homburg, Germany). Daily remote monitoring was initiated as scheduled for the next fifteen days. However, after five days of home treatment, several alarms were triggered during the peritoneal fluid drainage phases. Upon contacting the patient, a nurse learned that Mrs MS had been experiencing constipation for at least 3 days, accompanied by mild pain in the left iliac quadrant. Consequently, a decision was made to recommend a hospital visit. During the objective examination, the doctor observed a distended abdomen with present albeit slow peristalsis. Laboratory analysis showed a normal distribution of electrolytes with a serum creatinine level of 9.34 mg/dL and blood urea nitrogen (BUN) level of 45 mg/dL. The diagnosis of constipation was confirmed, and cathartic therapy was administered. During the hospital visit, the patient received additional training from a nurse on maintaining good food hygiene habits and managing gastroenteric problems. While waiting for the restoration of normal gut function, the peritoneal program was remotely adjusted, reducing the drain flow from 230 mL/min to 180 mL/min to improve the quality of the patient’s sleep. After one week of therapy, normal bowel activity was restored, allowing the doctors to remotely reinstate the initial peritoneal program.

### 2.3. Clinical Case 2

TS, a 72-year-old Caucasian gentleman, has end-stage kidney disease stemming from diabetic nephropathy. Since May 2017, he has been navigating the intricacies of peritoneal dialysis, specifically adhering to the APD protocol using the Homechoice Claria system (ClariaTM) and engaging in Sharesource monitoring, both provided by Baxter (Healthcare, Deerfield, IL, USA). In August 2021, Mr. TS proactively reached out to the medical center, expressing concerns about cloudy drains during nocturnal peritoneal dialysis sessions, although he did not report any associated abdominal pain. In response, he was promptly invited to the hospital, bringing with him the drained peritoneal fluids for closer examination. Upon a comprehensive objective evaluation, healthcare professionals observed mild diffuse pain in the abdomen upon applying pressure, yet no fever was noted. The peritoneal effluent displayed a mild cloudiness, and the exit site of the catheter exhibited no discernible signs of inflammation. The peritoneal effluent leukocyte count revealed 175 cells/mm^3^, prominently featuring 84% polymorphonuclear leukocytes (PMN). Further laboratory analysis divulged a white blood cell (WBC) count of 9800 cells/mm^2^, with 70% neutrophils, a hematocrit (Hct) level of 34%, a C-reactive protein (CRP) level of 17 mg/L, a procalcitonin (PCT) level of 3 ng/mL, a BUN level of 32 mg/dL and a serum creatinine level of 8.72 mg/dL. Promptly, a diagnosis of peritoneal dialysis-related peritonitis was established, a conclusion solidified three days later by positive results in the peritoneal effluent culture, specifically identifying S. epidermidis multisensible. It is worth noting that blood cultures yielded negative results. Subsequently, Mr. TS returned home, having undergone a modification of his dialysis prescription to continuous ambulatory peritoneal dialysis (CAPD). Additionally, he commenced empirical antibiotic therapy, initially receiving cefazolin and ceftazidime, with the latter being discontinued once the culture results were available.

Throughout the antibiotic regimen, diligent daily check-ins were conducted by the nursing team to remote monitor Mr. TS’s condition. Encouragingly, after three days, the effluent leukocyte count demonstrated a notable decrease to 76 cells/mm^3^, accompanied by 45% PMN. The antibiotic therapy continued for a total of fourteen days. As the treatment transitioned into a critical phase, the nursing team dedicated time to retraining Mr. TS on the meticulous management of his peritoneal dialysis catheter. This educational initiative aimed to empower the patient with the knowledge and skills necessary to prevent future occurrences of infections and ensure the ongoing success of his peritoneal dialysis treatment.

### 2.4. Clinical Case 3

AC, a 65-year-old woman diagnosed with ESKD due to hypertensive nephropathy, has been undergoing APD at home using the Sleep-safe Harmony system and Patient Card Unicard Reader. The patient initiated APD in January 2023. Routine RPM was scheduled as part of the center’s telemedicine protocol. In June 2023, during a routine telemedicine check, the nurse noticed a lack of data transmission for the past two consecutive days. Recognizing the importance of consistent data transmission for effective remote monitoring, the nurse promptly initiated a call to AC to investigate the issue. Upon reaching the patient, it was discovered that the APD machine was experiencing connectivity issues due to an error with the modem. AC was unaware of the problem and appreciated the nursing team’s intervention. The nurse guided the patient through a series of troubleshooting steps, and it became evident that a simple modem restart might resolve the issue. Following the modem restart, the APD machine successfully re-established the connection, and the missing data from the previous days were transmitted promptly. The nurse took this opportunity to reinforce the importance of routine machine checks and the need to address any technical issues promptly. The data sent confirmed the adequacy of dialysis and treatment. Subsequent remote monitoring sessions for AC proceeded without further connectivity problems (Table 2).

## 3. Discussion

In the realm of nephrology, the application of RPM in peritoneal dialysis has emerged as a transformative approach to patient care. Today, several studies indicate that this field is expanding [12,13,14,15,16], with new research flourishing in this regard, such as in the PDTAP study [17].

**Figure 1:**
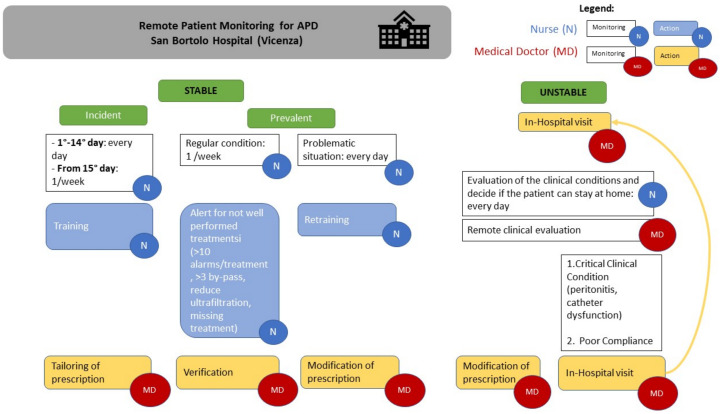
Workflow of our clinical approach for RM.

In our APD-RPM clinical model, we divide patients into incident and prevalent patients and into stable and unstable patients for better follow-up, management, and clinical adequacy for each clinical condition. In Figure 1, we report a schematic APD-RPM workflow for patient follow-up and for care team organization in clinical practice. In the case of incident patients, the nurses monitor all the dialysis treatments in the telemedicine platform during the first fifteen days after APD starts, then once a week. In the case of prevalent patients, the check is routinely performed once a week. For each patient, nurses spend between five and ten minutes evaluating the data. Furthermore, they report it to the physicians when the dialysis is not performed well: for example, if they find more than ten alarms per treatment, or if the patient performs more than three bypasses, or when the ultrafiltration is less than prescribed, or when certain treatments have not been performed. Moreover, the physicians and the nurses check the treatments in the platform whenever the patients call the PD center reporting a dialytic problem. After the platform evaluation, the physicians can remotely change the dialysis prescription if needed and give the patient some suggestions by phone, avoiding an unscheduled hospital visit if the clinical conditions are stable. Meanwhile, if the clinical conditions are unstable, such as in case of overhydration, peritonitis, or catheter dysfunction, the patient is invited to come to the hospital. In addition, in cases of non-compliance, such as treatments not being performed or more than three bypasses taking place, an in-person visit is required. Similarly, when the patient returns home after the solution of an acute episode, daily remote monitoring is scheduled for incident patients.

Our RPM protocol, implemented at the Vicenza Peritoneal Dialysis Center, represents a dynamic integration of technology into the management of peritoneal dialysis patients. The key to our protocol includes the real-time monitoring of APD treatments and the utilization of Internet-connected devices to transmit vital patient data to our healthcare facility. Moreover, the transition from traditional in-person visits to televisits, as endorsed by the World Health Organization’s guidelines [18], has been seamlessly integrated into our RPM protocol. As a result, our patients experience the dual benefits of maintaining the comfort of home-based treatment while receiving vigilant and efficient clinical oversight.

Ensuring patient adherence to the RPM protocol is a critical aspect and lot of studies have been performed to test successful implementation in peritoneal dialysis [19,20,21,22]. Our study witnessed a commendable level of patient compliance with the daily remote monitoring requirements. The high level of adherence can be attributed to the user-friendly nature of the RPM system. Historically, patients had to physically bring electronic cards containing treatment data to the PD center for review. However, with the advent of e-health devices, our RPM approach allows for the remote monitoring of APD treatments. This transition from the traditional “card system” to a digital, remote monitoring model significantly streamlined the process, enhancing overall adherence.

We reported our real experience with patients, reporting three examples in this case series and our strategy for implementing RPM in clinical practice. The integration of case reports serves as a pivotal component in elucidating the real-world efficacy of our RPM protocol in peritoneal dialysis. In the first case, we want to show a comprehensive approach to a common complication for incidental patients. This case highlights the RPM protocol’s role in not only optimizing APD performance but also in detecting and managing broader health issues. In the second case, we explain how our protocol works in a common acute condition. Daily remote check-ins during the antibiotic regimen showcased the protocol’s role in continuous monitoring and patient support, resulting in a successful resolution of the peritonitis episode. Using this case series report, we want to highlight this protocol’s ability to adapt to different clinical settings. Moreover, we aim to bridge the gap between theoretical protocol descriptions and practical applications. In this way, we offer valuable insights into the RPM protocol’s seamless integration into clinical workflows and its potential to enhance personalized patient care, ultimately leading to improved outcomes. It has been demonstrated that the RPM provides closer and prompter care of PD patients, improving the treatment tailoring and allowing early troubleshooting [10]. This management can prevent acute problems and in particular reduce urgent visits or hospitalization, leaving the patients at home as much as possible [23,24]. We can speculate that, as a consequence of the lower patient access to the PD center, telemedicine allows timesaving in clinical practice, at the same time enhancing patient-focused care. However, some concerns have to be focused on to ensure good clinical practice and widespread clinical acceptance and uptake. Firstly, the knowledge of these programs must be a part of physicians’ and nurses’ training, to strengthen care team innovation and capability [25]. The clinicians have to inform the patients and/or the caregivers about telemedicine, obtain informed consent, and educate them about the necessary procedures to send/receive the data to/from the PD center [26]. Furthermore, the traditional team workflow has to be changed to include the time dedicated to the treatment evaluation by nurses and physicians. Otherwise, in case of data overload and a lack of clinical contact with the patients, this important output could be lost or underestimated.

Finally, Walker et al., in their qualitative interview study, confirmed that, according to patients’ and caregivers’ expectations and experiences, RPM increases patients’ knowledge, enhances the partnership with clinicians, and improves timely access to treatment. However, it is also important to note that RPM does not replace the face-to-face relationship between patients and care teams [13].

Supported by our experience, we suggest that the care team should define a protocol that helps to obtain a correct approach to RPM, while considering available resources. The workflow has to consider the different needs of patients in order to assure frequent remote control for incident or unstable patients. Meanwhile, prevalent and stable patients can perform their home treatment more independently, helped by periodic and deferred clinical supervision.

One potential obstacle to our RPM is that several patients are elderly and may face challenges in managing certain technologies. Fortunately, this hurdle is easily overcome through patient training, facilitated by the automation of data transmission systems. Another commonly encountered issue is data transfer problems, often resolved after restoring the connection, as observed in Case Report 3.

While RPM proves to be a powerful tool in peritoneal dialysis management, the sheer volume of data generated daily poses a significant challenge. The continuous monitoring of APD treatments, combined with routine clinical parameters, results in an overflow of information that demands careful analysis. In our RPM protocol, we have implemented a structured approach to address this challenge. For stable patients, data analysis occurs four times a month, striking a balance between regular surveillance and avoiding the overwhelming task of daily scrutiny. This approach has proven effective in managing the flow of information and ensuring timely intervention when necessary. Implementing AI-driven data analysis can allow for more frequent and thorough examinations without imposing an impractical burden on healthcare professionals [27,28]. By setting up AI algorithms to identify and prioritize critical data points, the RPM protocol can achieve a higher sensitivity to deviations from the norm [29,30].

## 4. Conclusions

In the dynamic landscape of healthcare, the integration of remote patient monitoring (RPM) into peritoneal dialysis has proven to be a transformative force, redefining the boundaries of patient care. Our comprehensive exploration of the RPM protocol implemented at the Vicenza Peritoneal Dialysis Center highlights not only its effectiveness in managing patients but also its adaptability to the evolving needs of modern healthcare. We think that our workflow ensures robust patient follow-up and efficient organization of the care team. The presented case series confirms the efficacy of the RPM approach within our clinical practice. Crucially, at the heart of our RPM protocol is a commitment to a patient-centric approach. The seamless integration of technology into the lives of our patients allows for not only enhanced clinical oversight but also the preservation of the comfort and autonomy associated with home-based peritoneal dialysis. Through proactive interventions and personalized care, we strive to empower patients on their healthcare journey.

As we stand at the intersection of technology and patient care, the future horizons for remote patient monitoring in peritoneal dialysis appear promising. The ongoing research and implementation of AI-driven analyses hold the key to refining protocols and unlocking new dimensions of patient care. By continually embracing innovation and evidence-based practices, we can ensure that RPM evolves as a stalwart companion in the healthcare journey.

## Figures and Tables

**Table 1:** Remote patient monitoring (RPM) schedule and actions prompted by physicians and nurses.

Patients		APD Remote Monitoring			Actions Prompted by
		What	When	Who	Nurses
Stable	Incident	All the treatments	Every day for 15 days	Nurses and physicians	Training for patients to manage cycler
	Prevalent	All the treatments	Once a week	Nurses	Report poorly performed treatments to physicians
		Not well performed treatments	Every day	Nurses and physicians	Retraining in case of technical issues
Unstable	When patients stay at home after clinical evaluation in PD center	All the treatments	Every day	Nurses and physicians	Verify the treatments and call the patients

APD: automated peritoneal dialysis; PD: peritoneal dialysis.

**Table 2:** Clinical data for cases 1, 2 and 3.

	Case 1	Case 2	Case 3
Age; years	67	72	65
Sex	F	M	F
Cause of ESKD	IgA-nephropathy	Diabetic nephropathy	hypertensive nephropathy
Type of PD	APD	APD	APD
Program at home	Sleep-safe Harmony and the Patient Card Unicard Reader	Homechoice Claria system and engaging in Sharesource monitoring	Sleep-safe Harmony system and Patient Card Unicard Reader

## Data Availability

All data generated or analyzed during this study will be included in a future article. Further enquiries can be directed to the corresponding author.
